# Viruses in Vietnamese Patients Presenting with Community-Acquired Sepsis of Unknown Cause

**DOI:** 10.1128/JCM.00386-19

**Published:** 2019-08-26

**Authors:** Nguyen To Anh, Nguyen Thi Thu Hong, Le Nguyen Truc Nhu, Tran Tan Thanh, Chuen-Yen Lau, Direk Limmathurotsakul, Xutao Deng, Motiur Rahman, Nguyen Van Vinh Chau, H. Rogier van Doorn, Guy Thwaites, Eric Delwart, Le Van Tan

**Affiliations:** a Oxford University Clinical Research Unit, Ho Chi Minh City, Vietnam; b Centre for Tropical Medicine and Global Health, Nuffield Department of Medicine, University of Oxford, Oxford, United Kingdom; c Collaborative Clinical Research Branch, Division of Clinical Research, National Institute of Allergy and Infectious Diseases, National Institutes of Health, Bethesda, Maryland, USA; d Mahidol Oxford Tropical Research Unit, Bangkok, Thailand; e Hospital for Tropical Diseases, Ho Chi Minh City, Vietnam; f Blood Systems Research Institute, San Francisco, California, USA; g Department of Laboratory Medicine, University of California, San Francisco, California, USA; Memorial Sloan Kettering Cancer Center

**Keywords:** Vietnam, community-acquired sepsis, viral metagenomics

## Abstract

Community-acquired (CA) sepsis is a major public health problem worldwide, yet the etiology remains unknown for >50% of the patients. Here we applied metagenomic next-generation sequencing (mNGS) to characterize the human virome in 492 clinical samples (384 sera, 92 pooled nasal and throat swabs, 10 stools, and 6 cerebrospinal fluid samples) from 386 patients (213 adults and 173 children) presenting with CA sepsis who were recruited from 6 hospitals across Vietnam between 2013 and 2015. Specific monoplex PCRs were used subsequently to confirm the presence of viral sequences detected by mNGS. We found sequences related to 47 viral species belonging to 21 families in 358 of 386 (93%) patients, including viruses known to cause human infections. After PCR confirmation, human viruses were found in 52 of 386 patients (13.4%); picornavirus (enteroviruses [*n* = 14], rhinovirus [*n* = 5], and parechovirus [*n* = 2]), hepatitis B virus (*n* = 10), cytomegalovirus (*n* = 9), Epstein-Barr virus (*n* = 5), and rotavirus A (*n* = 3) were the most common viruses detected. Recently discovered viruses were also found (gemycircularvirus [*n* = 5] and WU polyomavirus, Saffold virus, salivirus, cyclovirus-VN, and human pegivirus 2 [HPgV2] [*n*, 1 each]), adding to the growing literature about the geographic distribution of these novel viruses. Notably, sequences related to numerous viruses not previously reported in human tissues were also detected. To summarize, we identified 21 viral species known to be infectious to humans in 52 of 386 (13.4%) patients presenting with CA sepsis of unknown cause. The study, however, cannot directly impute sepsis causation to the viruses identified. The results highlight the fact that it remains a challenge to establish the causative agents in CA sepsis patients, especially in tropical settings such as Vietnam.

## Abstract (precis)

Community-acquired (CA) sepsis is a major public health problem worldwide, yet the etiology remains unknown for >50% of the patients. Here we applied metagenomic next-generation sequencing (mNGS) to characterize the human virome in 492 clinical samples (384 sera, 92 pooled nasal and throat swabs, 10 stools, and 6 cerebrospinal fluid samples) from 386 patients (213 adults and 173 children) presenting with CA sepsis who were recruited from 6 hospitals across Vietnam between 2013 and 2015.

## INTRODUCTION

According to the WHO, approximately 30 million cases of sepsis with 6 million deaths occur globally each year (1). Approximately 70% of sepsis cases are attributed to community-acquired (CA) infections (1). The increasing frequency of antimicrobial resistance and the diversity of pathogens (including bacteria and viruses) that may cause CA sepsis further complicate current diagnostic efforts, in turn posing challenges to patient management (2). Indeed, despite extensive laboratory investigations, the causes of a substantial proportion of cases of CA sepsis remain unknown. In a recent etiological study of 1,578 patients with CA sepsis, conducted by the Southeast Asia Infectious Disease Clinical Research Network, the etiology (viruses, bacteria, and parasites) was established for only 48% (3). While this diagnostic yield is comparable to that of previous reports, the unknown etiology for >50% of the patients may be attributed to the low sensitivity of current diagnostic tests and/or the diversity of the causative agents that may be responsible for this important clinical condition. Furthermore, Southeast Asia is one of the major hot spots for the emergence of novel pathogens, as illustrated by the emergence of Nipah virus, severe acute respiratory syndrome (SARS) coronavirus, avian influenza virus A (H5N1), avian influenza virus A (H7N9), enterovirus A71 (EV-A71), and, more recently, Zika virus (4, 5).

Metagenomic next-generation sequencing (mNGS) has emerged as an unbiased, sequence-independent method for the detection of pathogens, especially viruses, in clinical samples (6–13). Using mNGS, we previously discovered a novel cyclovirus (cyclovirus-Vietnam [cyclovirus-VN]) in 4% of Vietnamese patients presenting with central nervous system (CNS) infections, although the pathogenicity and natural hosts of this virus remain unresolved (8, 14).

Improving our knowledge about the causative agents of CA sepsis can inform clinical management, while active surveillance for novel pathogens in this region is of public health significance. In this study, we use mNGS to characterize the viral contents of clinical samples collected from patients enrolled in an etiological study of sepsis of unknown etiology across Southeast Asia between 2013 and 2015 (3).

## MATERIALS AND METHODS

### Clinical specimens and patient data.

**FIG 1:**
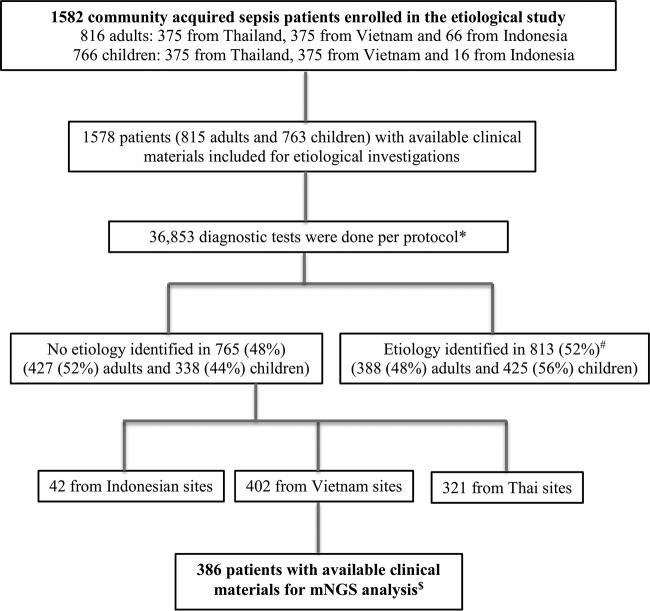
Flow chart showing an overview of the diagnostic output of the original study. *, see the original study (3) and Table S1 in the supplemental material for more details; ^#^, the causative agents detected are detailed in the report of the original study (3); ^$^, more details about the analysis of those 386 patients can be found in Fig. 2.

The clinical specimens and patient data used for mNGS analysis were derived from an etiological study of CA sepsis conducted at multiple hospitals across Indonesia (*n* = 3), Thailand (*n* = 4), and Vietnam (*n* = 6) between 2013 and 2015 (3). Hospitalized patients with suspected or documented CA infections, fulfilling the diagnostic criteria for sepsis of the 2012 Surviving Sepsis Campaign (adults) (15) or the definitions of the Pediatric Sepsis Consensus Conference (16), were enrolled within 24 h of admission (3). A total of 1,582 patients were enrolled (750 each from Vietnam and Thailand; 82 from Indonesia) (Fig. 1). Per the study protocol, serum samples were collected from all patients; additional samples, including pooled nasal and throat swabs, cerebrospinal fluid (CSF), and stools, were collected when clinically indicated. After collection, all clinical samples were stored at –80°C. Additionally, information about the demographics, clinical entities, and outcomes of the patients was retrieved from a publicly available data set of the original study that was deposited at https://figshare.com/articles/Data_set_-_Causes_and_outcomes_of_sepsis_in_southeast_Asia_a_multinational_multicentre_cross-sectional_study_NCT02157259_/3486866/1.

**FIG 2:**
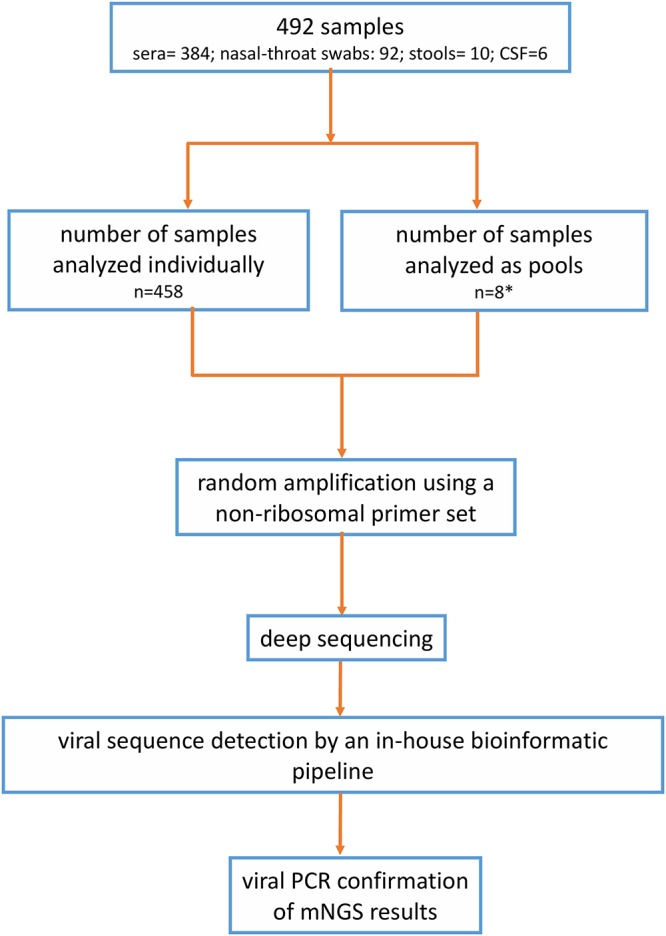
Flow chart showing how samples were analyzed. *, includes 4 pools of 5 samples, 3 pools of 4 samples, and 1 pool of 2 samples.

Of 749 patients from Vietnam, 402 (54%) had no etiology identified via extensive clinical and reference laboratory workups in the original study (Fig. 1; see also Table S1 in the supplemental material); of these, 386 (96%) had clinical materials available for additional etiological investigation and were thus included for viral metagenomic analysis in this study (Fig. 1) (3). In total, 492 samples (6 CSF samples, 92 pooled nasal and throat swabs, 384 serum samples, and 10 stool samples) from these 386 patients with sepsis of unknown etiology were included in the analysis. Due to the availability of the materials, most samples were analyzed individually (*n* = 458) or in pools of multiple samples (*n* = 8) (Fig. 2).

### Sample pretreatments and NA isolation.

Prior to nucleic acid (NA) isolation, 100 μl of clinical sample was treated with 2 U/μl of Turbo DNase (Ambion, Life Technology, Carlsbad, CA, USA) and 0.4 U/μl RNase I (Ambion) at 37°C for 30 min. Viral NA was then isolated from nuclease-treated materials using a QIAamp viral RNA kit (Qiagen GmbH, Hilden, Germany) and was recovered in 50 μl of elution buffer.

### dsDNA synthesis and sequencing.

Double-stranded DNA (dsDNA) was synthesized from isolated viral NA using a set of 96 nonribosomal random primers (17), amplified by PCR, and sequenced on an Illumina MiSeq platform (Illumina, San Diego, CA, USA) as described previously (18, 19). In brief, 10 μl of extracted viral NA was converted to dsDNA using FR26RV-Endoh primers (19), SuperScript III enzyme (Invitrogen, Carlsbad, CA, USA), RNaseOUT (Invitrogen), exo-Klenow fragment (Ambion, Life Technology, Carlsbad, CA, USA), and RNase H (Ambion). Subsequently, the synthesized dsDNA was randomly amplified using the FR20RV primer (5′-GCCGGAGCTCTGCAGATATC-3′). The random PCR product obtained was then purified with the use of Agencourt AMPure XP beads (Beckman Coulter) and was quantified with a Qubit dsDNA HS (high-sensitivity) kit (Invitrogen). Finally, 1 ng of purified product was subjected to library preparation using a Nextera XT sample preparation kit (Illumina) and was sequenced using a MiSeq reagent kit, v3 (600 cycles) (Illumina), on a MiSeq platform (Illumina).

### mNGS data analysis.

The mNGS data were analyzed using an in-house viral metagenomic pipeline running on a 36-node Linux cluster to identify the presence of viral sequences in the tested specimens as described previously (20). In brief, after duplicate reads and reads belonging to human or bacterial genomes were filtered out, the remaining reads were assembled *de novo*. The resulting contigs and singlet reads were then aligned against a customized viral proteome database using a BLAST (Basic Local Alignment Search Tool)-based approach. Next, the candidate viral reads were aligned against a nonredundant nonvirus protein database to remove any false-positive reads (i.e., reads with expected [E] values higher than those against viral protein databases). Any virus-like sequence with an E value of ≤10^−5^ was considered a significant hit. Finally, a reference-based mapping approach was employed to assess the levels of identity and genome coverages of the corresponding viruses.

### PCR confirmation of viral reads.

Because of the focus of the present study, specific PCRs were used to confirm the mNGS hits for viral species that are known to be infectious to humans and for recently discovered viruses that have been reported in human tissues previously but remain of uncertain tropism. Depending on the availability of the clinical materials, virus-specific PCRs were carried out either on leftover NA after mNGS experiments or on newly extracted NA. An mNGS result was considered positive only if it was subsequently confirmed by a corresponding viral PCR analysis of original NA materials derived from the corresponding individual samples. All PCR primers and probes used were either derived from previous publications or newly designed based on the sequences generated by mNGS (see Table S2 in the supplemental material).

### Phylogenetic analysis.

Sequence alignment and phylogenetic tree reconstructions of the sequences obtained were carried out using ClustalW alignment and maximum likelihood methods available within Geneious 8.1.5 (Biomatters) and IQ-TREE (21), respectively.

### Ethical statement.

The study was reviewed and approved by the Institutional Review Boards of collaborating hospitals in Vietnam and the Oxford Tropical Research Ethics Committee (OxTREC), University of Oxford, Oxford, United Kingdom.

### Accession number(s).

The metagenomics data obtained in this study have been deposited in GenBank, and the accession numbers can be found via BioProject accession number PRJNA526981.

## RESULTS

### Demographics, clinical features, and outcomes for patients with sepsis of unknown origin.

The baseline characteristics and 28-day mortality data of all patients (including the 386 patients included in the mNGS analysis) from Vietnamese sites enrolled in the original study are presented in Table 1. Retrospectively, 129 (34.4%) adult patients (including 54 of the 213 with undiagnosed cases [25%]) had SOFA (Sequential Organ Failure Assessment) scores of ≥2, fulfilling the diagnostic criteria presently used for sepsis in adults as defined by Sepsis-3 (22). For pediatric sepsis, no harmonized criteria similar to those for sepsis in adults have been established (23).

**TABLE 1:** Demographic and clinical data for CA sepsis patients

Characteristic	No. (%) of patients:					
	Included in mNGS analysis^*a*^			Not included in mNGS analysis		
	Total (*n* = 386)	Adults (*n* = 213)	Children	Total (*n* = 363)	Adults (*n* = 162)	Children
			(*n* = 173)			(*n* = 201)
Male gender	224 (58)	122 (57.3)	102 (59)	204 (56)	84 (41)	120 (59)
Age						
<12 mo	NA	NA	45 (26)	NA	NA	75 (37.3)
≥1 to <5 yr	NA	NA	100 (57.8)	NA	NA	106 (52.7)
≥5 to <18 yr	NA	NA	28 (16.2)	NA	NA	20 (10)
≥18 to <40 yr	NA	94 (44.1)	NA	NA	68 (42)	NA
≥40 to <60 yr	NA	67 (31.5)	NA	NA	60 (37)	NA
≥60 yr	NA	52 (24.4)	NA	NA	34 (21)	NA
Geographic location						
North Vietnam	123 (32)	68 (32)	55 (32)	127 (35)	57 (35)	70 (34)
Central Vietnam	141 (37)	79 (37)	62 (36)	108 (30)	46 (28)	62 (31)
South Vietnam	122 (32)	66 (31)	56 (32)	128 (35)	59 (37)	69 (34)
SOFA score^*b*^						
≤1	NA	159 (75)	NA	NA	87 (53.7)	NA
≥2	NA	54 (25)	NA	NA	75 (46.3)	NA
Clinical presentation^*c*^						
Respiratory infection	158 (41)	97 (45)	61 (36)	212 (58)	70 (43)	142 (71)
Diarrhea	36 (9)	25 (12)	11 (6)	15 (4)	10 (6)	5 (2)
CNS infection	40 (10.5)	8 (4)	32 (18)	42 (12)	14 (9)	28 (14)
Systemic infection	152 (39.5)	83 (39)	69 (40)	94 (26)	68 (42)	26 (13)
28-day mortality						
Yes	10 (2.6)	8 (3.7)	2 (1)	16 (4)	9 (5)	7 (3)
No	373 (96.6)	203 (95.3)	170 (98)	337 (93)	149 (92)	188 (94)
Unknown	3 (<1)	2 (1)	1 (<1)	10 (3)	4 (3)	6 (3)

a NA, not applicable.

b Available for adult patients only.

c Defined on the basis of major clinical symptoms. Acute respiratory infection was defined as the manifestation of at least one respiratory symptom for no longer than 14 days. Acute diarrhea was defined as diarrhea for no longer than 14 days. Acute CNS infection was defined as the manifestation of CNS symptoms for no longer than 14 days or the presence of signs of CNS infection on admission. Systemic infection was defined as the absence of acute respiratory infection, acute diarrhea, and acute CNS infection.

There was considerable homogeneity between the group of patients included and the group not included in the mNGS analysis (Table 1). Among the 386 patients with sepsis of unknown cause whose data were included in the mNGS analysis, the most frequent clinical entity was acute respiratory infection (*n* = 158 [41%]), followed by systemic infection (*n* = 152 [39.5%]), diarrhea (*n* = 36 [9.3%]), and central nervous system (CNS) infection (*n* = 40 [10.5%]) (Table 1) (3). Ten of these patients (8 adults and 2 children) were recorded as deceased by day 28, accounting for 2.6% of total patients.

### Overview of virus-like sequences detected by mNGS.

In total, 466 samples were sequenced in five MiSeq runs, generating a total of >26 million reads (median reads per sample, 432,682; range, 540 to 1,916,732) (see Fig. S1 in the supplemental material). Despite the inclusion of a nuclease digestion step prior to NA isolation, viral reads accounted for only a small proportion of total reads, ranging from 168,028 (2.5%) to 287,307 (8.4%) reads/run. Evidence of sequences related to 47 viral species belonging to 21 families was detected in 358/386 (93%) patients. The viruses detected included those known to cause human infections, those with unknown pathogenicity, and viruses that have been reported previously to be contaminants found in mNGS data sets or that have not been reported in human samples, as detailed below. Additionally, codetection of ≥2 viruses in the same samples/patients was recorded for 13 patients (see Table S3). None of the 10 fatal cases had a viral etiology identified by mNGS.

**(i) Detection of viruses known to cause human infections.** NA sequences of 21 viral species known to be infectious to humans were detected in 137 of 466 (29%) clinical samples from 125 of 386 (32%) individuals by viral metagenomics. The detection rate was reduced to 13.4% (52/386) of the 386 patients included in the mNGS analysis after specific PCR confirmation. There was a significant difference in the number of viral reads generated by mNGS between the groups of samples that were subsequently found to be PCR positive or negative (see Fig. S2 in the supplemental material), while the total numbers of reads obtained were similar for the two groups (median [range], 493,794 [11,076 to 1,203,206] versus 461,486 [16,470 to 1,770,372]) (*P* = 0.58). The number of reads per sample in the group of samples in which viruses were found by mNGS and subsequently confirmed by PCR was significantly higher than that in the group in which no virus was found (median [range], 493,794 [11,076 to 1,203,206] versus 365,974 [540 to 1,916,732]) (*P* = 0.004), suggesting that the diagnostic yield of mNGS is dependent on the sequencing depth (i.e., the number of reads generated per sample).

**FIG 3:**
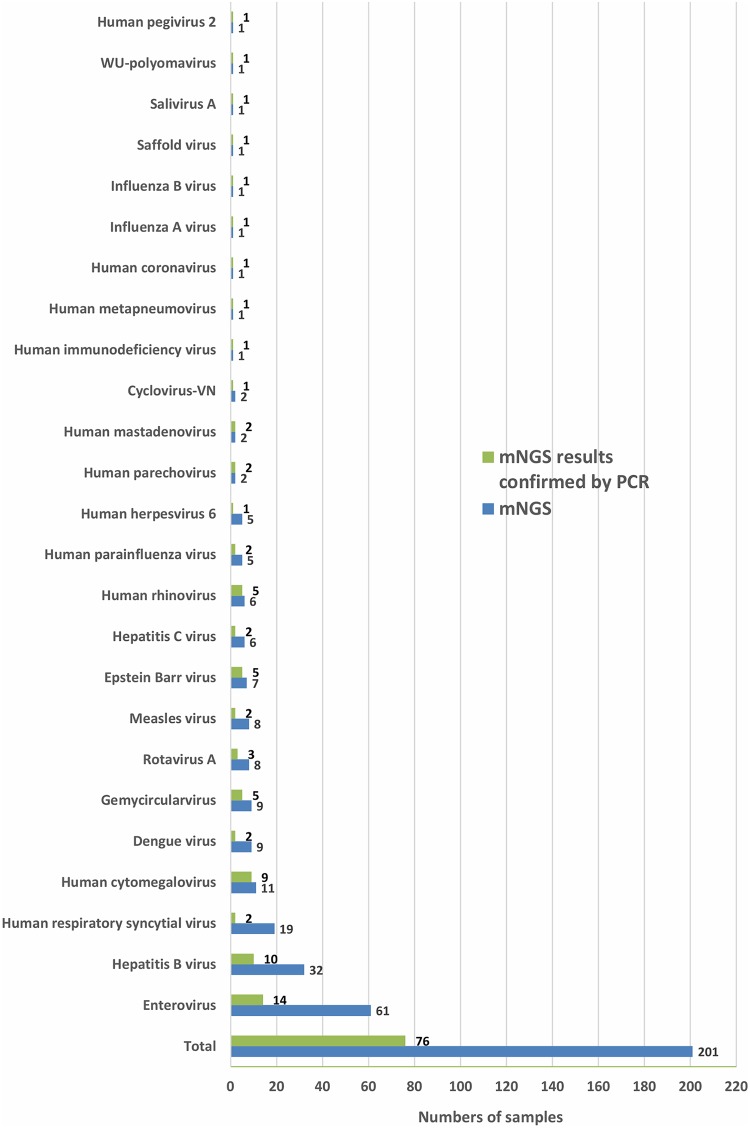
Bar chart showing the numbers of viruses known to be infectious to humans or previously reported in human tissues that were detected by mNGS, followed by PCR confirmation testing.

Of the viruses detected, enterovirus (EV) was the most common (14/386 [3.6%]), followed by hepatitis B virus (HBV) (9/386 [2.3%]), cytomegalovirus (CMV) (9/386 [2.3%]), human rhinovirus (HRV) (5/386 [1.3%]), Epstein-Barr virus (EBV) (5/386 [1.3%]), and rotavirus (3/386 [0.7%]) (Fig. 3). Detailed information about the numbers of viral reads and genome coverage is summarized in Table S7 in the supplemental material.

**(ii) Detection of sequences related to viruses with unknown pathogenicity.** Sequences related to four recently discovered viruses (gemycircularviruses, WU polyomavirus, human pegivirus 2 [HPgV-2], and cyclovirus-VN) whose pathogenicity or tropism remains unknown, but whose genetic materials have been reported in human samples previously, were identified by mNGS in 3.4% of the samples from the 386 patients included in the mNGS analysis. After specific PCR testing, the confirmed proportion of positive patients was reduced to 2.1% (5/386 [1.3%] had gemycircularvirus, and 1/386 [0.26%] each had WU polyomavirus, HPgV2, or cyclovirus-VN) (Fig. 3). Additionally, anellovirus-like sequences were found in the majority of the samples tested (362/466 [77%]), while sequences related to human pegivirus 1 and human papillomaviruses were found in 4/466 (<1%) and 1/466 (<1%) samples, respectively. Because these viruses are common nonpathogenic infectious agents, they were not subjected to subsequent PCR confirmation testing.

**(iii) Detection of sequences related to contaminants and/or viruses not previously reported in human samples.** Sequences related to common contaminants of mNGS data sets (including a parvovirus-like hybrid virus [24] and Kadipiro virus [25]) were detected in 96 and 5 samples, respectively (see Table S4 in the supplemental material). Additionally, sequences related to numerous viruses that have not been reported in human tissues previously were also found (Table S4). Here we focus our analysis on viruses that have been reported in human tissues.

### Viral detection by mNGS followed by PCR confirmation testing in different sample types.

**FIG 4:**
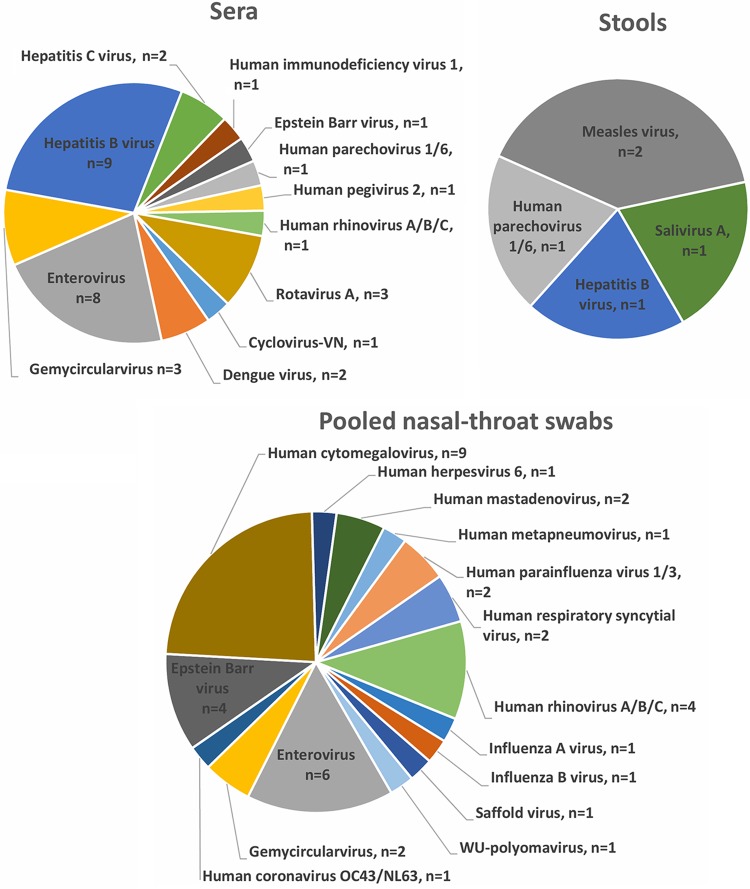
Numbers of viruses detected by mNGS, and then confirmed by virus-specific PCR, in clinical samples of different types.

The detection rates for human viruses or viruses reported in human tissues were 8% (32/384) for sera, 41% (38/92) for nasal-throat swabs, and 50% (5/10) for stool samples, while all 6 CSF samples available from 40 patients presenting with CNS infection were negative. More viruses were found in pooled nasal-throat swabs than in samples of other types (Fig. 4).

In the sera tested, 12 different viral species were detected, including the well-established human pathogens HBV (*n* = 9), EV (*n* = 8), rotavirus A (*n* = 3), dengue virus (DENV) (*n* = 2), hepatitis C virus (HCV) (*n* = 2), human parechovirus (*n* = 1), HRV (*n* = 1), and human immunodeficiency virus (HIV) (*n* = 1) (Fig. 4).

### Viral detection in different patient groups and clinical entities by mNGS followed by PCR confirmation testing.

**FIG 5:**
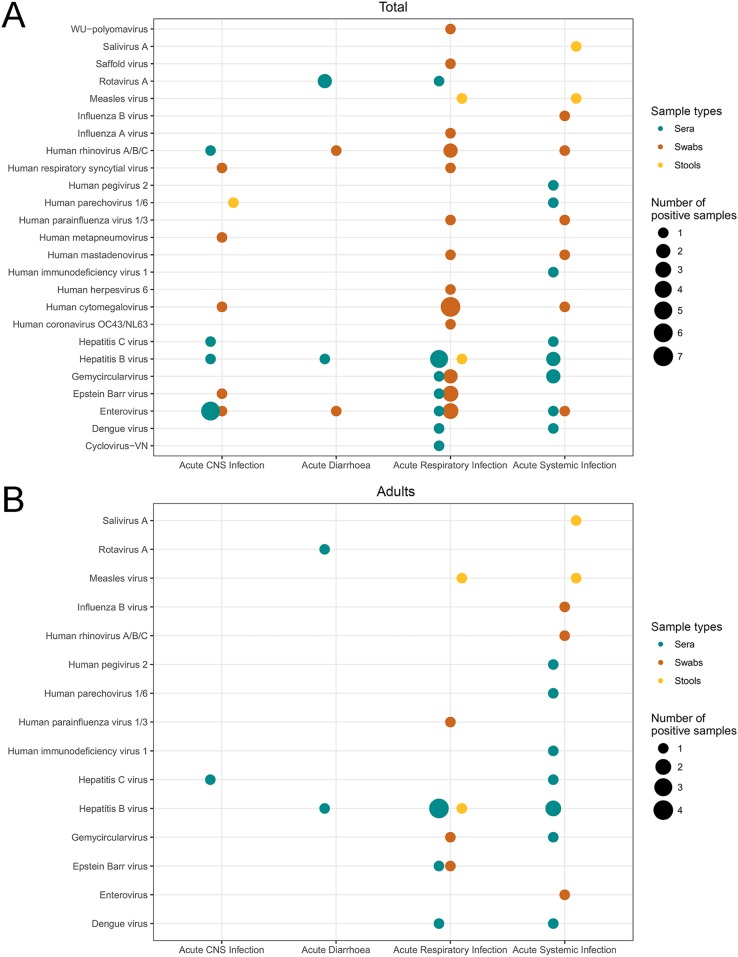

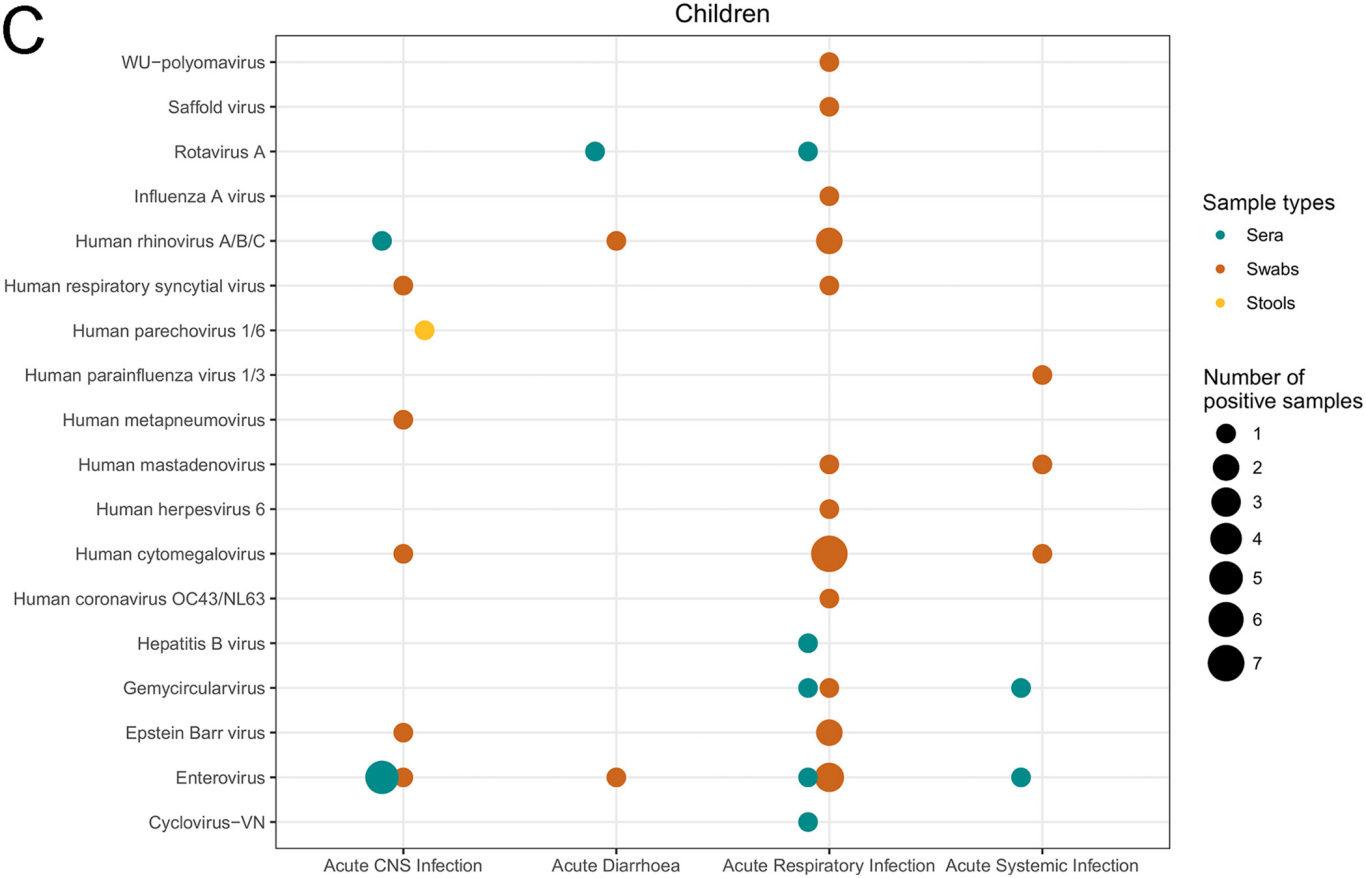
Numbers of viruses detected by mNGS, and then confirmed by virus-specific PCR, in different patient groups and clinical entities. Symbols are color-coded by sample type. (A) All patients included in the mNGS analysis; (B) adults; (C) children.

The frequencies of different viral species detected in different clinical entities and patient groups are shown in Fig. 5 and Fig. S3 in the supplemental material.

Regardless of the clinical sample type, the highest proportion of distinct viral infections was recorded in patients presenting with CNS infections (15/40 [37.5%]), followed by patients with respiratory infections (37/158 [23%]) and patients with systemic infections (19/152 [12.5%]). Of the 54 adults with a SOFA score of ≥2, 6 had a virus identified (from 2 samples with measles virus or HBV and 1 each with dengue virus, rotavirus A, gemycircularvirus, salivirus A, or EBV) (see Table S5 in the supplemental material).

Among the patients presenting with CNS infections, picornaviruses were the most common viruses detected (see Table S6 in the supplemental material); these included enterovirus, accounting for 7 of 15 (47%) viruses detected (6 in sera and 1 in a pooled nasal-throat swab), and HRV, detected in a serum sample. Rotavirus, a well-known cause of diarrhea, was detected in the blood of three diarrhea patients (two children and one adult).

In terms of age groups, EV and other respiratory viruses (e.g., respiratory syncytial virus [RSV] and HRV) were detected more frequently in children than in adults (Fig. 5). In contrast, blood-borne viruses (HIV, HCV, and HBV) were found more often in adults than in children (Fig. 5). Parechovirus, an established cause of pediatric infections, was detected in one adult presenting with a systemic infection.

### Genetic characterization of EV and HBV.

Excluding anellovirus-related sequences, mNGS generated sufficient sequence data for informative genetic characterization and phylogenetic inference of EV and HBV in 14 samples, including seven complete viral capsid protein 1 (VP1) sequences of enterovirus and seven complete HBV genomes. Phylogenetically, all seven EVs were classified into six different serotypes of enteroviruses A and B (echovirus 3, echovirus 6, echovirus 9, echovirus 16, coxsackievirus A2, and coxsackievirus A6), while the HBV strains belonged to genotypes B and C (see Fig. S4 and S5 in the supplemental material), supporting reports about circulating enterovirus serotypes and HBV genotypes in Vietnam (26–28).

For other viruses, due to the small numbers of genomic sequences recovered (two for DENV, two for gemycircularvirus, and one each for RSV, influenza B virus, HCV, measles virus, WU polyomavirus, and cyclovirus-VN), similar phylogenetic inference was deemed uninformative.

## DISCUSSION

We present the results of mNGS for exploration of the human virome in 386 patients presenting with CA sepsis of unknown cause who were enrolled in a multicenter observational study across Vietnam from 2013 to 2015. We identified 21 viral species known to be infectious to humans in 52 (13.4%) of 386 patients presenting with CA sepsis of unknown cause. The study, however, cannot directly impute sepsis causation involving the viruses identified. More specifically, on several occasions, viral detection in nonsterile materials, such as respiratory samples (including EBV and CMV) and stool samples, may simply reflect the carriage of such viruses in those bodily compartments rather than a clinical association. Similarly, viral detection (e.g., enterovirus) in the blood of patients with asymptomatic infections has been reported previously (29). Additionally, the detection of blood-borne viruses, such as HBV, HIV, and HCV, in serum samples might represent underlying diseases and not the causative pathogens leading to the hospital admission, although the detection of HIV RNA in a serum sample of a patient presenting with systemic infection may suggest an acute HIV infection. However, together with the clinical and epidemiologic data, the results present a provocative argument for a wide range of viral pathogens that might be associated with CA sepsis in Vietnam.

Epidemiologically, our results support previous findings regarding the frequent detection of common viruses in corresponding clinical entities and age groups. For example, we found rotavirus only in patients with acute diarrhea and RSV and viruses of the *Picornaviridae* family (HRV and EV) mostly in children. Additionally, we detected parechovirus in the blood of an adult presenting with acute systemic infection. Parechoviruses are a well-known cause of disease in children, ranging from acute gastrointestinal/respiratory infections to meningitis, but have increasingly been reported to cause infections in adults (30).

Nonpolio enteroviruses, such as EV-A71 and EV-D68, have become serious global threats. In fact, EV-A71 has overwhelmed countries of the Asia-Pacific region (including Vietnam) with large outbreaks of severe hand-foot-and-mouth disease since 1997 (31, 32). Recently, EV-D68 has emerged and caused large outbreaks of respiratory infections in the United States; this virus is epidemiologically linked with acute flaccid myelitis (33). The data presented here, together with the results of the original report (3), expand our knowledge about the clinical burden posed by nonpolio enteroviruses (HRV and particularly diverse EV serotypes) and parechoviruses in Vietnam.

mNGS detected several recently discovered viruses (Saffold virus, salivirus A, WU polyomavirus, gemycircularvirus, and HPgV-2), representing their first detection in Vietnam and adding to the growing literature about the geographic distribution of these newly identified viruses. Salivirus A has been linked to gastrointestinal infection, and Saffold virus has been reported in gastrointestinal and respiratory infection patients (34–37). Saffold virus has also been reported to be associated with myocarditis and aseptic meningitis (38, 39). Additionally, using a mouse model, studies have shown the neurotropic potential of Saffold virus (39–41). The pathogenicity of WU polyomavirus, gemycircularvirus, and HPgV-2 remains unresolved. Likewise, it is imperative to conduct follow-up studies to determine whether the detected sequences that are related to viruses not previously reported in human tissues are derived from other sources and whether the respective viruses are infectious to humans.

The results of the present investigation also emphasize the utility of serum samples for assessing the etiology of sepsis. Indeed, viruses of the families *Picornaviridae* (enterovirus, rhinovirus, and parechovirus), *Flaviviridae* (DENV), and *Caliciviridae* (rotavirus) were detected by mNGS in the sera included in this study. Notably, as per the design of the original etiological study, sera were not tested for these viruses by PCR (3). Likewise, while it remains unknown why the original study failed to detect common causes of respiratory/enteric infections (influenza A virus, influenza B virus, EV, etc.) in pooled nasal swabs by multiplex PCR assays (3), a slightly lower sensitivity of the multiplex PCR assays used than that of the respective monoplex PCR assays has been reported elsewhere (42).

Virus detection by mNGS is based on the detection of matching viral reads regardless of their number or the resulting genome coverage. While few metagenomic studies published to date have reported the use of specific PCR to verify metagenomic results subsequently, the failure of virus-specific PCR to confirm the original mNGS detections for many patients in the present study may be a consequence of cross talk (bleedthrough) contamination occurring as part of the sequencing procedure, a well-documented phenomenon (10, 43, 44). An alternative explanation is the low sensitivity, likely attributed to nucleotide mismatches, of some of the PCR primers used to confirm infection.

The absence of human viral pathogens in 87% of 386 patients may be attributed to the low sensitivity of our mNGS approach, especially in cases where the number of reads obtained was supposedly insufficient (Fig. S1 in the supplemental material), as suggested by the difference in the number of reads obtained between the groups of samples with and without a virus identified. Clearly, future research should address the question of what level of sequencing depth mNGS-based approaches need to achieve in order to reach the required sensitivity while maintaining cost-effectiveness. It is equally important to identify the factors (e.g., sample types and library preparation/sequencing methods) that may affect sequencing depth (i.e., the number of reads obtained) and assay sensitivity. Additional possibilities include the presence of the sepsis pathogen in nonanalyzed tissues, the presence of nonviral pathogens (e.g., bacteria and parasites) in tested specimens, and/or the inclusion of patients with no infection (e.g., those with conditions caused by toxicity whose clinical presentations mimic infections) in the study.

In summary, we report the application of mNGS for patients presenting with CA sepsis of unknown etiology. Our results highlight challenges in identifying possible viral culprits in patients with CA sepsis and show that diverse viral agents might be responsible for such devastating conditions in tropical settings such as Vietnam. Therefore, rigorous testing for a wide range of viral pathogens in samples from different body compartments collected early after symptom onset, when viral loads are usually highest, is likely to have the greatest yield. Under these circumstances, mNGS is a promising approach because of its capacity to simultaneously detect and genetically characterize viral pathogens in patient samples without the need for prior knowledge of genomic information about the targeted pathogens, thus enhancing the ability to identify infectious etiologies of sepsis and facilitating optimal targeted management.

## Supplementary Material

Supplemental file 1
