# Supplementary material for: Viruses in Vietnamese Patients Presenting with Community-Acquired Sepsis of Unknown Cause
Source: J Clin Microbiol. 2019 Aug 26;57(9):e00386-19. doi: 10.1128/JCM.00386-19 (PMC6711913; doi:10.1128/JCM.00386-19)
Supplement: Supplemental file 1 [file JCM.00386-19-s0001.pdf]

SUPPLEMENTARY MATERIALS FOR

Viruses in Vietnamese patients presenting with community acquired sepsis of unknown cause

Nguyen To Anh[1], Nguyen Thi Thu Hong[1], Le Nguyen Truc Nhu[1], Tran Tan Thanh[1], Chuen-Yen Lau[3], Direk Limmathurotsakul[4], Xutao Deng[6], Motiur Rahman[1], Nguyen Van Vinh Chau[5], H. Rogier van Doorn[1,2], Guy Thwaites[1,2], Eric Delwart[6] and Le Van Tan[1], for the Southeast Asia Infectious Disease Clinical Research Network*

Author affiliations

[1]Oxford University Clinical Research Unit, Ho Chi Minh City, Vietnam (NTA, NTTH, LNTN, TTT, HRvD, MR, GT, LVT)

[2]Centre for Tropical Medicine and Global Health, Nuffield Department of Medicine, University of Oxford, Oxford, UK (DL, HRvD, GT)

[3]Collaborative Clinical Research Branch, Division of Clinical Research, National Institute of Allergy and Infectious Diseases, National Institutes of Health, Bethesda, USA (CYL)

[4]Mahidol Oxford Tropical Research Unit, Bangkok, Thailand (DL)

[5]Hospital for Tropical Diseases, Ho Chi Minh City, Vietnam (NVVC)

[6]Blood Systems Research Institute, San Francisco, CA, USA (DF, DX, ED)

Department of Laboratory Medicine, University of California, San Francisco, CA, USA (DF, DX, ED)

Correspondence: Nguyen To Anh, Oxford University Clinical Research Unit, Ho Chi Minh City, Vietnam; Tel: (+84 28) 8384009; Fax: (+84 8) 9238904; E-mail: anhnt@oucru.org

Le Van Tan, Oxford University Clinical Research Unit, Ho Chi Minh City, Vietnam; Tel: (+84 28) 8384009; Fax: (+84 8) 9238904; E-mail: tanlv@oucru.org

Word count: abstract: 249, full text: 3445

Keywords: community acquired sepsis, viral metagenomics, Vietnam

Running title: Metagenomic NGS of sepsis patients of unknown cause

Supplementary Figure 1: Bar chart showing the number of reads obtained from individual samples. Each vertical bar represents one sample.

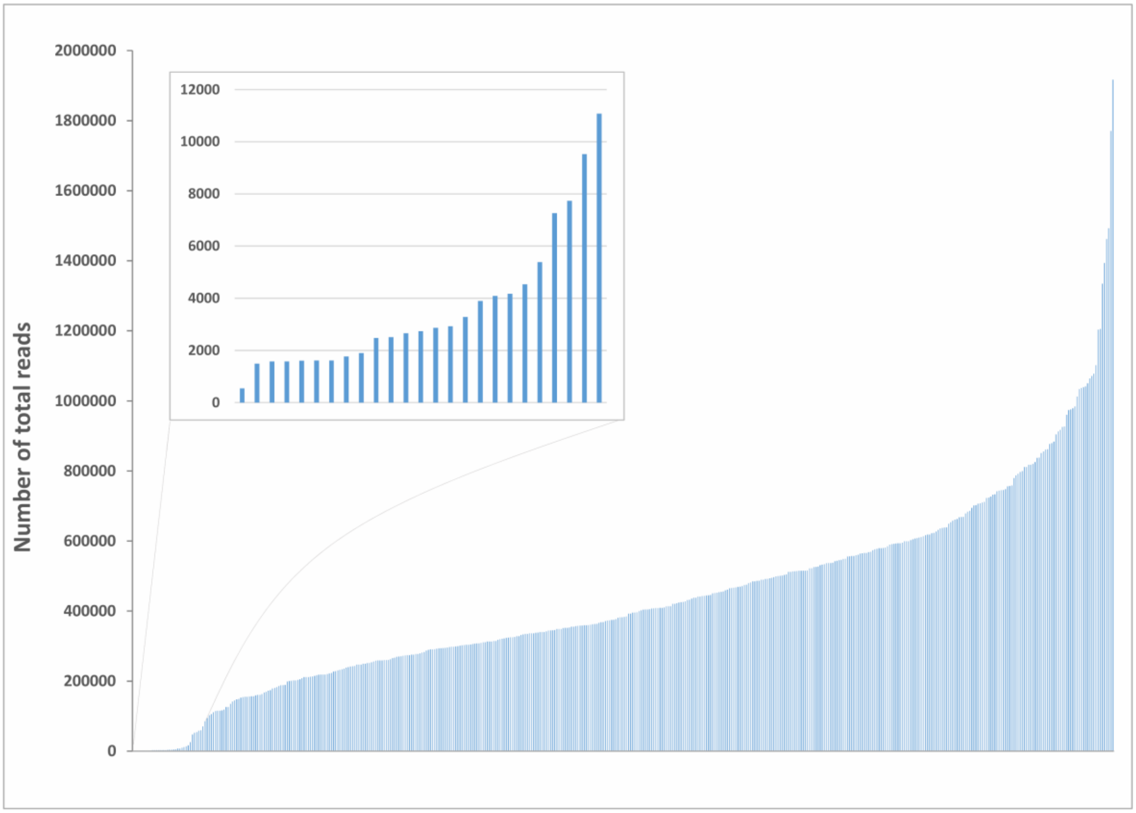

Supplementary Figure 2: Boxplots showing the difference in the numbers of viral hits between PCR positive and negative groups

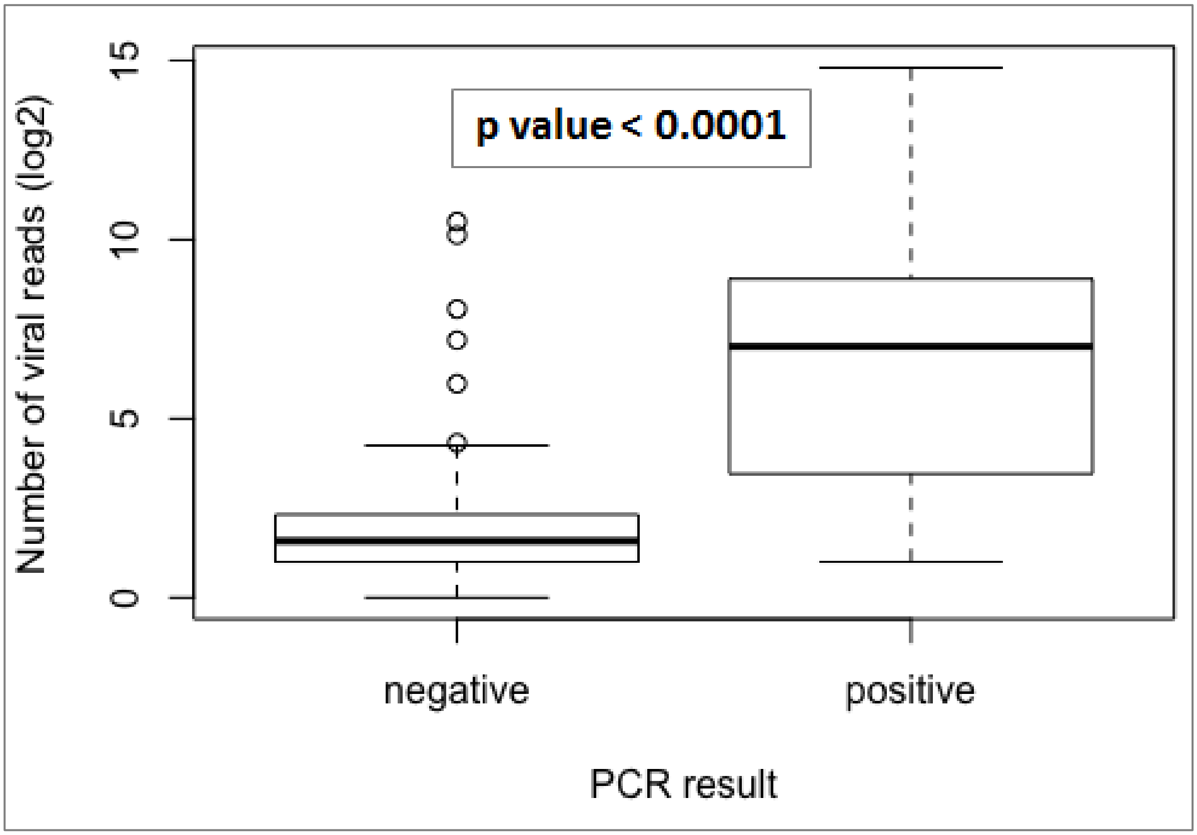

Supplementary Figure 3: Viral detection by mNGS, which were then confirmed by viral specific PCR, in different clinical entities

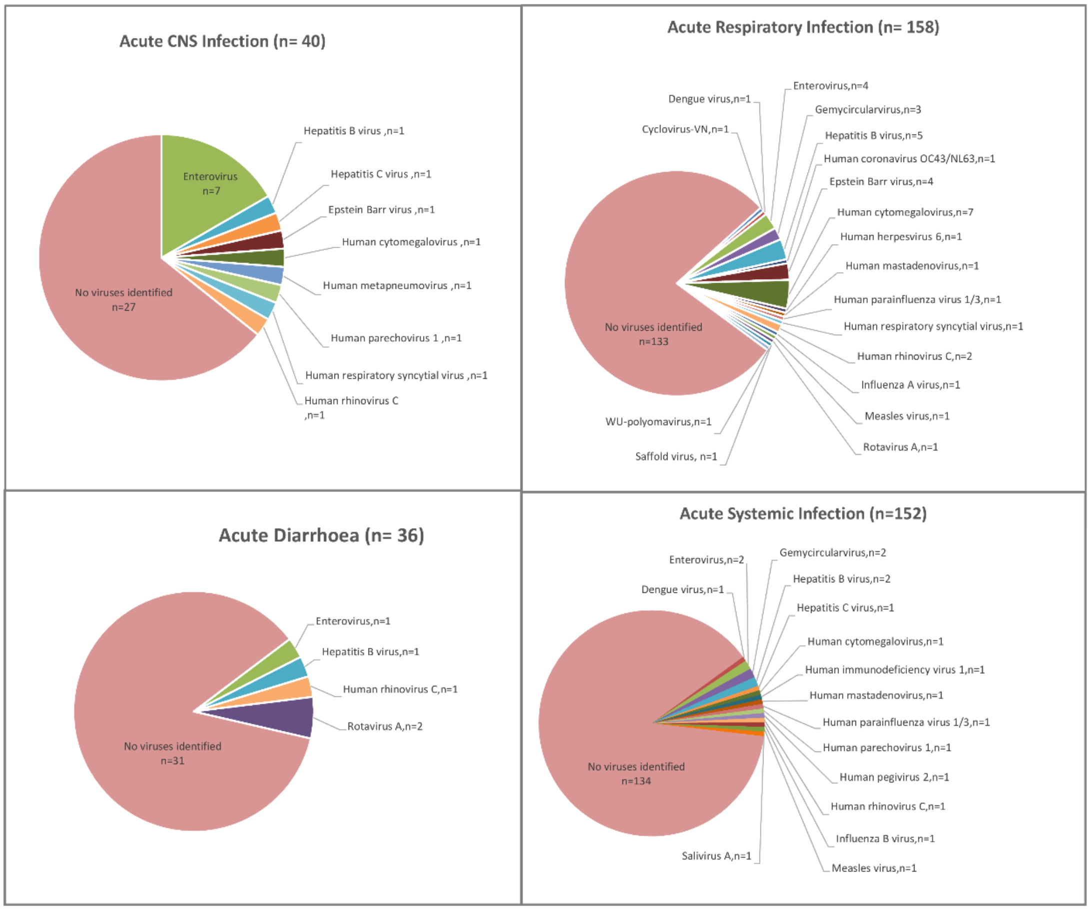

Supplementary Figure 4: Maximum Likelihood tree based on complete VP1 sequences of different enterovirus serotypes illustrating the relatedness between enterovirus serotypes recovered in the present study by mNGS (blue triangles) and representative EV serotypes

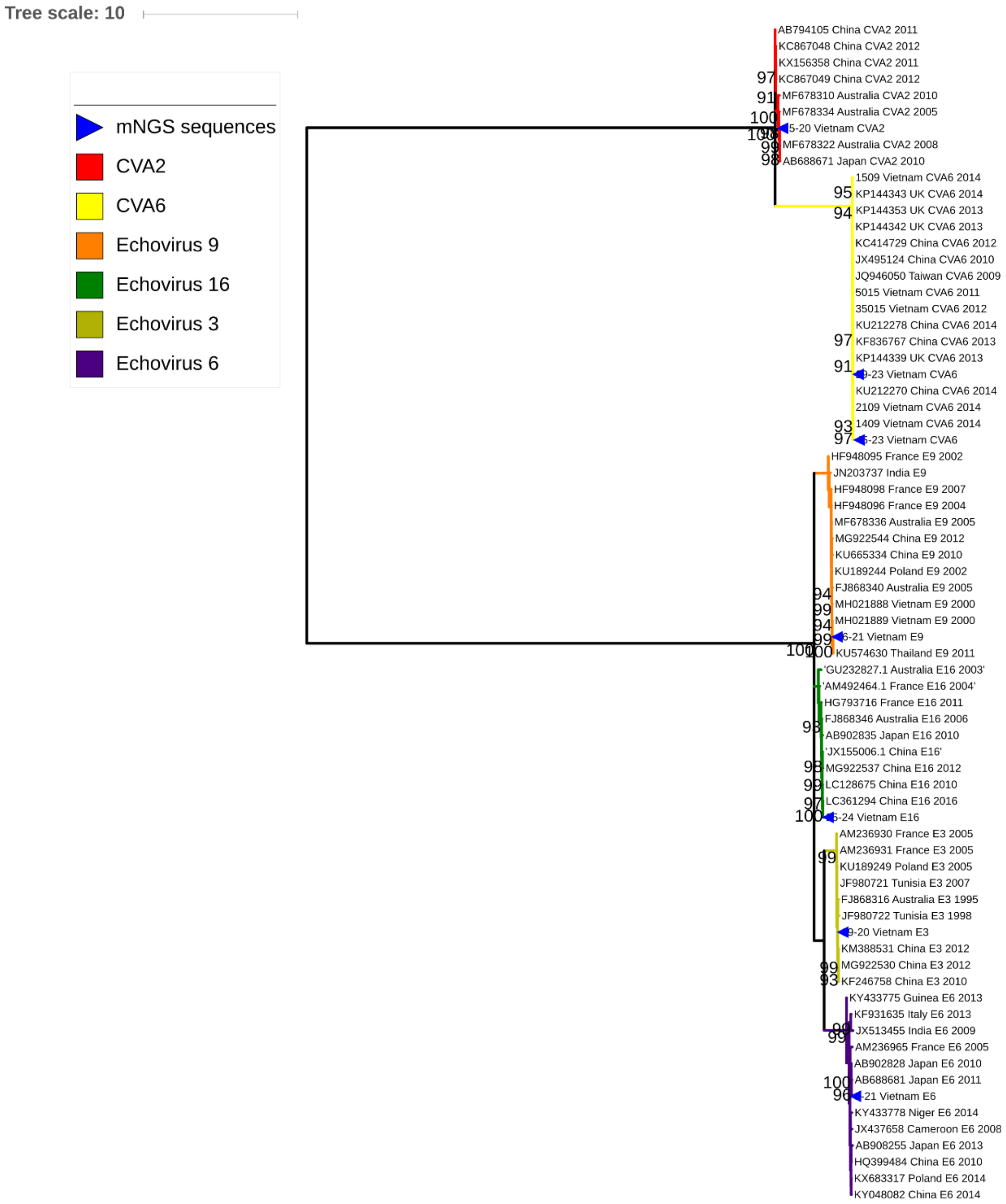

Supplementary Figure 5: Complete coding sequence based Maximum Likelihood tree showing the relationship between HBV sequences recovered in the present study (black triangles) and representative HBV genotypes

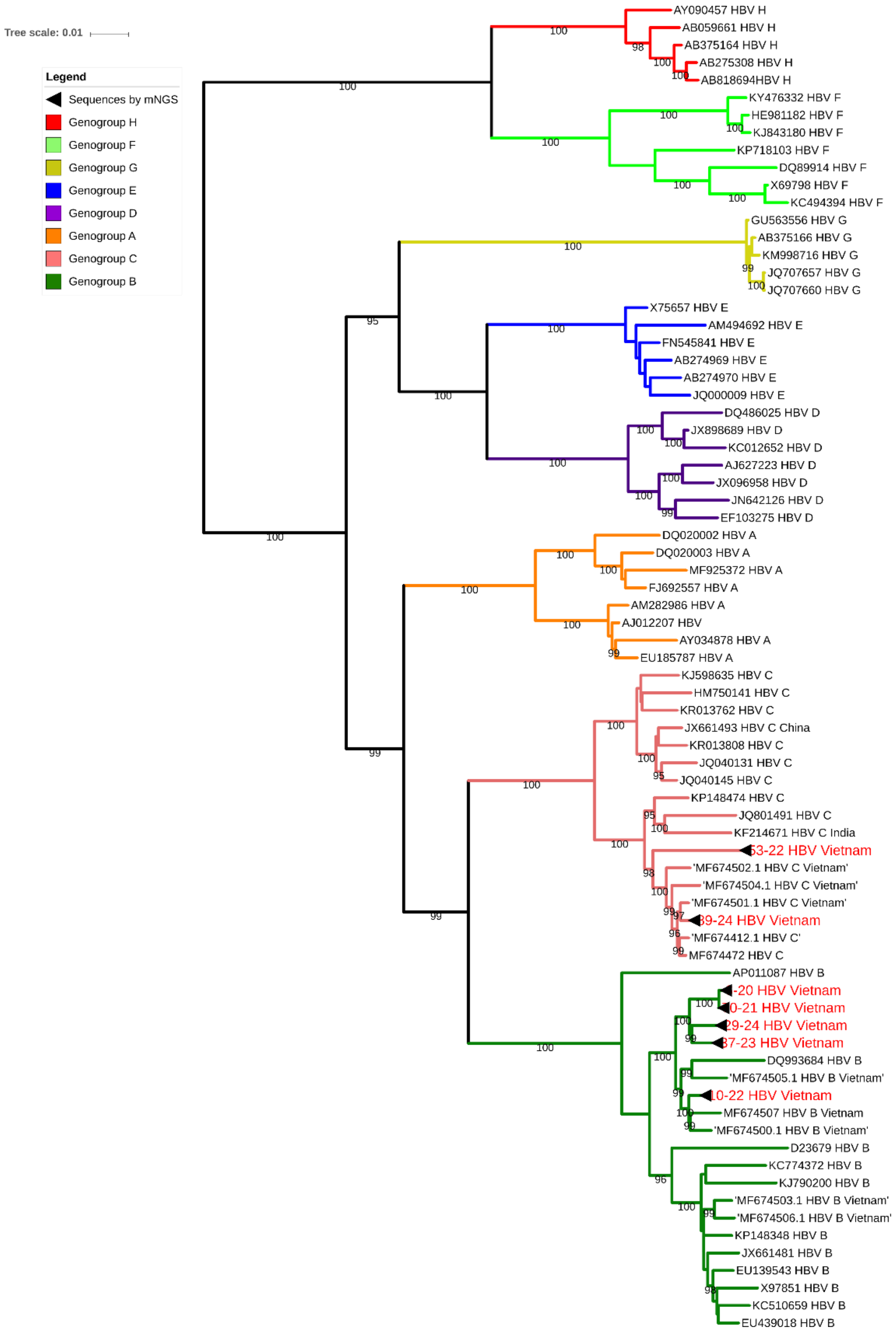

Supplementary Table 1: Diagnostic work-up carried out as per the study protocol of the original report

**Pathogens[#, **]**	**Assay #1 and samples**	**Assay #2 and samples**	**References**
Leptospirosis	Whole-blood PCR	Microagglutination tests of paired sera	[1, 2]
Scrub typhus	Whole-blood PCR	IFA of paired sera	[3, 4]
Rickettsiosis and murine typhus	Whole-blood PCR		[4, 5]
Murine typhus	IFA of paired sera		[3]
Bacteraemia	Whole-blood PCR		[6, 7]
Hantavirus	Serum PCR		[8]
Japanese encephalitis virus	CSF ELISA		[9]
Mumps	CSF ELISA		Mumps virus (Parotitis) IgM ELISA Kit (IBL International, Germany)
Measles	CSF ELISA		Measles virus IgM micro-capture ELISA (IBL International, Hamburg, Germany)
Rubella	CSF ELISA		Rubella virus IgM micro-capture ELISA (Novatec Immundiagnostica Technologie & Waldpark, Germany)
Dengue	CSF PCR		[10]
Herpes simplex virus 1 and 2	CSF PCR		[11]
Varicella-zoster virus	CSF PCR		[12]
Enterovirus	CSF PCR		[13]
Parechovirus	CSF PCR		[14]
N. meningitidis	CSF PCR		[15]
S. pneumoniae	CSF PCR		[15]

H. influenza type b	CSF PCR		[15]
S. suis	CSF PCR		[16]
Respiratory viruses	Pooled nasal-throat swab PCR		[14]
Respiratory bacteria	Pooled nasal-throat swab PCR		[17–19]
Adenovirus	Stool PCR		[20]
Astrovirus	Stool PCR		[21]
Norovirus	Stool PCR		[22]
Rotavirus	Stool PCR		[22]

Notes to Supplementary Table 1:

#Diagnostic tests performed in every case as part of standard of care at participating hospitals including complete blood count, blood culture, urine culture, gram/ZN smears, and sputum culture if patients have respiratory symptoms, stool examination and stool culture if patients have diarrheal symptoms and CSF examination and CSF culture if patients have neurological symptoms and CNS infection is suspected.

**Diagnostic tests performed in every case per study protocol including dengue RDT (NS1 and IgM, Standard Diagnostics, South Korea), influenza RDT (QuickVue, Quidel Corporation, USA), only for paediatric patients age < 7 years old and leptospirosis RDT (Leptospira IgM/IgG, Standard Diagnostics), only for paediatric patients age ≥ 7 years old and all adult patients

*Four multiplex real-time PCR assays detecting 15 virus subtypes of 10 viruses; Influenza (A & B), Adenovirus, Enterovirus, Respiratory syncytial virus (A & B), Metapneumovirus, Rhinovirus, Parainfluenza virus (1, 2, 3 & 4), Coronavirus, Bocavirus (subtype OC43 & NL63), and Parechovirus

$5 real-time assays detecting 5 bacteria; *Legionella pneumophila*, *Mycoplasma pneumoniae*, *Chlamydophila pneumoniae*, *Chlamydophila psittaci* and *Bordetella pertussis*

Supplementary Table 2: List of primers and probes used for subsequent PCR confirmation experiments

Viruses	Oligo sequence (5'-3')			Sources
	Forward	Reverse	Probe	
Measles	ATTACATCAGGATCCGG	GTATTGGTCCGCCTCATC		[23]
HBV	GGACCCCTGCTCGTGTTACA	GAGAGAAGTCCACCMCGAGTCTAGA	FAM-TGTTGACAARAATCCTCACAATACCRCAGA-TAMRA	Newly designed
Rotavirus	ACC ATC TWC ACR TRA CCC TC	GGT CAC ATA ACG CCC CTA TA	FAM-ATG AGC ACA ATA GTT AAA AGC TAA CAC TGT CAA-BHQ1	[24]
Enterovirus	CCCTGAATGCGGCTAAT	ATTGTCACCATAAGCAGCC	CY5-ACCCAAAGTAGTCGGTTCCG -BHQ3	[25]
Dengue	AAGGACTAGAGGTTAGAGGAGACCC	CGTTCTGTGCCTGGAATGATG	FAM- AACAGCATATTGACGCTGGGAGAGACCAGA-BHQ1	[26]
Dengue 2	CCATACACGCCAAACATGAA	GGGATTTCCTCCCATGATTCC	FAM-AGGGTGTGGATTCGAGAAAACCCATGG-BHQ1	[27]
HIV1	GGTGCGAGAGCGTC	ATGCTRTCATCATYTCTTC		[28]
	ATGGGTRAARGTARTAGAAGAAAAG GG	CTGCCTGRTGYCCYCCCACTA		
HCV	AGACTGCTAGCCGAGTAGYGTTGG	TGCTCATGDTGCACGGTCTACGA	FAM-TTGTGGTACTGCCTGATAGGGTGCTT -BHQ1	Newly designed
PIV 1	ATCTCATTATTACCYGGACCAAGTCT ACT	CATCCTTGAGTGATTAAGTTTGATGA ATA	CYAN500-AGGATGTGTTAGAYTACCTTCATTATCAATTGGTGATG-DB	[29]
PIV2	CTGCAGCTATGAGTAATC	TGATCGAGCATCTGGAAT	LCRED610-AGCCATGCATTCACCAGAAGCCAGC-BBQ	[29]
PIV3	ACTCTATCYACTCTCAGACC	TGGGATCTCTGAGGATAC	LCRED670-AAGGGACCACGCGCTCCTTTCATC-BBQ	[29]
PIV4	GATCCACAGCAAAGATTCAC	GCCTGTAAGGAAAGCAGAGA	HEX-TATCATCATCTGCCAAATCGGCAA-BHQ1	[29]
Coronavirus OC43	GGTGGYTGGGAYGATATGTTACG	KRTTTGGCATAGCACGATCACA	6-FAM-ATGTTGACAAYCCTGTWCTTATGGGTTGGG-MGBNFQ	[29]
Coronavirus NL63	GCTRAGCATGATTTCTTTACTTGG	CARTYTTKTTCATCAAAGTTACGCA	6-FAM-CAGARTCATTTATGGTAATGTTAGTAGACA-MGBNFQ	[29]
PEV	GGGTGGCAGATGGCGTGCCATAA	CCTRCGGGTACCTTCTGGGCATCC		[30]
	YCACACAGCCATCCTCTAGTAAG	GTGGGCCTTACAACTAGTGTTTG		
Rhinovirus	AGSCTGCGTGGCKGCC	ACACGGACACCCAAAGTAGT	CYAN500-TCCTCCGGCCCCTGAATGYGGCTAAYC-DB	[29]
MPV	AGCTTCAGTCAATTCAACAGAAG	CCTGCAGATGTYGGCATGT	LCRED670-TGTTGTGCGGCAGTTTTCAGACAATGC-BBQ	[29]
FA	GACAAGACCAATCCTGTCACYTCTG	AAGCGTCTACGCTGCAGTCC	LCRED610-TTCACGCTCACCGTGCCCAGTGAGC-BBQ	[29]
FB	TCGCTGTTTGGAGACACAAT	TTCTTTCCCACCGAACCA	CYAN500-AGAAGATGGAGAAGGCAAAGCAGAACT-DB	[29]
RSV	ATGAACAGTTTAACATTACCAAGT	GTTTTGCCATAGCATGACAC	LCRED610-TGACTTCAAAAACAGATGTAAGCAGCTCC-BBQ	[29]
			LCRED610-TTATGACATCAAAAACAGACATAAGCAGCTCAG-BBQ	
ADV	CAGGACGCCTCGGRGTAYCTSAG	GGAGCCACVGTGGGRTT	LCRED670-CGGGTCTGGTGCAGTTTGCCCGC-BBQ	[29]
Saffold virus	CTAATCAGAGGAAAGTCAGCAT	GACCACTTGGTTTGGAGAAGCT		[31]
	CAGCATTTTCCGGCCCAGGCTAA	GCTATTGTGAGGTCGCTACAGCTGT		
Salivirus	CCCTGCAACCATTACGCTTA	CACACCAACCTTACCCCACC		[32]
	ATTGAGTGGTGCAYGTGTTG	ACAAGCCGGAAGACGACTAC		Newly designed

Wu-polyomavirus	TGTTACAAATAGCTGCAGGTCAA	GCTGCATAATGGGGAGTACC		[33]
Human herpesvirus 6	TTTGCAGTCATCACGATCGG	AGAGCGACAAATTGGAGGTTTC		[34]
Human herpesvirus 4	GAGGAATTGCCCTTGCTATT	CCTTAGTGGGCCAGGTTGT	FAM -TCGTCTCCCCTTTGGAATGGC-TAMRA	Newly designed
Human herpesvirus 5	CCAAGCGGCCTCTGATAACCA	GGTCATCCACACTAGGAGAGCAGA	FAM-ATGAAGCGCCGCATTGAGGAGATCT-TAMRA	[35]
Gemycircularvirus	GTGGTAATGGTCGTCGGTATTC	CCTCATCATTCGTAGTAAGCAATCTCA		[36]
	AGTCCTGAATGTTTCCACTCG	CAAGCGTTCCCTCGAAAATGAC		Newly designed
Cyclovirus VN	GAGCGCACATTGAAAGAGCTAAA	TCTCCTCCTTCAATGACAGAAACAAC	FAM-CGADAATAAGGMATACTGCTCTAAAGSTGGCG-BHQ1	[37]
Human pegivirus 2	CGCTGATCGTGCAAAGGGATG	GCTCCACGGACGTCACACTGG	CY5-GCACCACTCCGTACAGCCTGAT-BHQ2	[38]

Supplementary Table 3: Detection of ≥2 viruses in the same samples/patients

Detected in	Serum	Pooled swabs	Stool
1 Adult	HBV		HBV and Measles virus
1 Child		Enterovirus, Influenza A and Cytomegalovirus	ND
1 Child	Cyclovirus VN and Gemycircularvirus	Cytomegalovirus	ND
1 Child		Enterovirus and Human rhinovirus A	ND
1 Child		Enterovirus and Human Herpesvirus 4	ND
1 Child	Enterovirus	Cytomegalovirus and Human herpesvirus 4	ND
1 Adult	Human immunodeficiency virus, Hepatitis C virus and Human Pegivirus 2	ND	ND
1 Adult	HBV and Dengue	ND	ND
1 Adult	ND	ND	Measles and Salivirus A
1 Child	ND	Cytomegalovirus and Human respiratory syncytial virus	ND
1 Child	ND	Cytomegalovirus and Human mastadenovirus	ND
1 Child	ND	Human herpesvirus 6 and Saffold virus	ND
1 Child	ND	Enterovirus and Human metapneumovirus	ND

Note to Supplementary Table 6: ND: not detected

Supplementary Table 4: List of common contaminants and viruses not reported in human samples

Viral family	Species	Genome	Number of matching reads	Best BLASTx E value	Detected in (n)	Other virus found	Patient group	Related species was previously reported in	References
Adenoviridae	Bovine mastadenovirus C	dsDNA	4	3.47E-12	Serum (1)		Adults	Cattle	[39]
Coronaviridae	Bulbul coronavirus HKU11	ssRNA	2	2.92E-06	Serum (1)		Adults	Wild bird	[40]
Coronaviridae	Penaeus monodon circovirus VN11	ssRNA	2,5&15	9.46E-05	Pooled swabs (n=1)<br>Serum (2)		Children and Adults	Numerous including pigs	[41]
Nodaviridae	Nodamura virus	ssRNA	2	9.13E-11	Stool (1)	Shuangao insect virus 11	Adults	Insects	[42]
Picornaviridae	Boone cardiovirus	ssRNA	17	5.45E-07	Serum (1)		Children	Rats	[43]
Picobirnaviridae	Dromedary picobirnavirus	dsRNA	8	4.90E-87	Pooled swabs (1)		Adults	Camels	[44]
Parvoviridae	Bat parvovirus	ssDNA	3	1.22E-08	Serum (2)		Children	Bat	[45]
Papillomaviridae	Human papillomavirus	circular dsDNA	4	2.86217E-42	Serum (1)		Adult		
Parvoviridae	Densovirus	ssDNA	≤ 559	5.31243E-96	Pooled swabs (12)<br>Serum (38)<br>CSF (1)		Children and Adults	Mosquitoes	[46]
Partitiviridae	Partitivirus	dsRNA	≤ 152	2.0824e-103	Pooled swabs (4)<br>Serum (22)		Children and Adults	Fungi	[47]
Parvoviridae	Parvovirus NIH-CQV	ssDNA	≤ 104	2.43E-52	Pooled swabs (4)<br>Serum (87)<br>Stool (1)<br>CSF (3)		Children and Adults	Qiagen column contaminant	[48]
Reoviridae	Kadipiro virus	dsRNA	3	7.87E-32	Serum (5)		Adults and Children	Contaminant	[49, 50]

Reoviridae	Lutzomyia reovirus 1	dsRNA	24	5.54E-07	Serum (1)		Adults	Sand flies	[51]
Reoviridae	Eubenangee virus	dsRNA	1	9.23E-05	Serum (1)	Tilligerry virus	Adults	Marsupials, cattle, mosquitoes and *Culicoides*	[52]
Reoviridae	Cypovirus	dsRNA	1,4,7	4.03196E-40	Serum (3)		Children and Adults	Insect	[53]
Rhabdoviridae	Curionopolis virus	ssRNA	6	4.45E-05	Serum (1)		Children	Culicoides	[54]
Totiviridae	Saccharomyces cerevisiae virus L-BC (La)	dsRNA	9	7.45478E-21	Pooled swabs (1)<br>Serum (3)		Children and Adults	Fungi	[55]
Totiviridae	Scheffersomyces segobiensis virus L	dsRNA	≤ 857	3.50723E-118	Pooled swabs (3)<br>Serum (12)<br>Stool (2)<br>CSF (1)		Children and Adults	Fungi	[56]
Unclassified	Magnaporthe oryzae RNA virus	RNA	9,14,15& 21	1.06135E-39	Serum (4)		Children and Adults	Fungi	[57]
Unclassified	Mosquito VEM virus SDRBAJ	ssDNA	3	1.40E-07	Serum (1)	CRESS virus	Adults	red snapper tissue	Unpublishe d paper
Unclassified	Nepavirus	ssDNA	2	6.44E-06	Serum (1)		Children	Untreated Sewage	[58]

Supplementary Table 5: Viral species found in (different body compartments of) adult patients with a SOFA score of ≥2

Patients	Sera	Pooled nasal and throat swabs	Stool
1	Rotavirus A	ND	ND
2	Hepatitis B virus	ND	Measles and hepatitis B virus
3	Dengue	ND	ND
4	Gemycircularvirus	ND	ND
5	ND	Epstein-Barr virus	ND
6	ND	ND	Measles and Salivirus A

Note to Supplementary Table 5: ND: not detected

Supplementary Table 6: The frequency of six most common viruses detected by mNGS in different clinical entities

Clinical presentation	Enterovirus (N=14)	Hepatitis B virus (N=9)	Cytomegalovirus (N=9)	Rhinovirus (N=5)	Epstein-Barr virus (N=5)	Rotavirus (N=3)
**CNS infection, n (%)**	7 (50)	1(11.1)	1(11.1)	1(20)	1(20)	0
**Respiratory infection, n (%)**	4 (28.6)	5(55.6)	6(66.7)	2(40)	4(80)	0
**Diarrhea, n (%)**	1(7.1)	1(11.1)	0	1(20)	0	3(100)
**Systematic infection, n (%)**	2(14.3)	2(22.2)	2(22.2)	1(20)	0	0

Supplementary Table 7: The number of viral reads and genome coverage in individual samples

Virus	Sample type	Number of total reads	Number of viral reads	E-value	Percentage of genome coverage (contig length/genomic size, bp)
Cyclovirus VN	Serum	442730	17	1.43937E-53	90.0% (1,671/1,856)
Cytomegalovirus	Swabs	493794	9	2.51E-38	>1% (624/235,403)
Cytomegalovirus	Swabs	211254	25	4.4231E-66	>1% (1,046/235,272)
Cytomegalovirus	Swabs	504418	200	1.4765E-109	1.3% (2,861/223,782)
Cytomegalovirus	Swabs	591732	12	2.90191E-52	>1% (900/235717)
Cytomegalovirus	Swabs	855702	4	5.94419E-25	>1% (309/235,272)
Cytomegalovirus	Swabs	1101990	1629	0	20.1% (44,960/223,782)
Cytomegalovirus	Swabs	1203206	53	3.20843E-51	1.9% (4,517/236,032)
Cytomegalovirus	Swabs	533616	86	8.87413E-53	2.2% (5,402/235,834)
Cytomegalovirus	Swabs	126026	204	4.7457E-111	7.1% (15,891/223,782)
Dengue virus	Serum	427286	3828	0	94.9% (10,174/10,723)
Dengue virus	Serum	560500	4721	0	95.0% (10,188/10,723)
Enterovirus	Serum	565748	405	0	42.0% (3,077/7,328)
Enterovirus	Serum	861012	6536	8.5729E-57	97.8% (6,795/6,946), 26.4% (1,941/7,345)
Enterovirus	Serum	348940	22	9.1753E-53	27.9% (1,841/6,606)
Enterovirus	Swabs	711594	329	0	31.4% (2,318/7,345)
Enterovirus	Swabs	493794	11	8.23215E-53	8% (579/7,345bp)
Enterovirus	Swabs	876786	29	1.5706E-155	61.8% (4,390/7,104)
Enterovirus	Serum	443284	170	5.827E-180	45.5% (3,379/7,434)
Enterovirus	Swabs	904408	6	2.31739E-22	2% (146/7,206)
Enterovirus	Swabs	825274	787	0	48.5% (3,204/6,612)
Enterovirus	Serum	172824	131	1.5905E-169	19.1% (1,421/7,432)
Enterovirus	Serum	200880	14	7.2996E-36	7.2% (537/7,427)
Enterovirus	Serum	349020	166	0	13.2% (980/7,433)
Enterovirus	Serum	205366	184	0	10.7% (703/6,591)
Enterovirus	Swabs	102766	5	1.30753E-39	2% (127/7,368)
Epstein-Barr virus	Serum	755526	4	2.59398E-36	<1% (336/169,864)
Epstein-Barr virus	Swabs	604874	3	1.98845E-32	1.6% (2,789/169,864)
Epstein-Barr virus	Swabs	904408	2	4.13442E-11	1.6% (2,795/169,864)
Epstein-Barr virus	Swabs	732950	2	1.18234E-12	1.5% (2,607/169,864)
Epstein-Barr virus	Swabs	126026	6	3.45686E-24	1.7% (2,905/169,864)
Gemycircularvirus SL1	Serum	381902	1668	3.06932E-60	100% (2,199)
Gemycircularvirus SL1	Swabs	883776	23	1.4508E-112	77.2% (1,697/2,199)
Gemycircularvirus SL1	Serum	442730	41	1.49764E-60	3.8% (85/2,199)
Gemycircularvirus SL1	Serum	281200	11	1.649E-102	52.5% (1,156/2,199)
Gemycircularvirus SL1	Swabs	219956	2	1.79015E-49	22.5% (494/2,199)
Hepatitis B virus	Serum	11076	183	5.56485E-51	94.7% (3,044/3,215)
Hepatitis B virus	Stool	441248	127	1.074E-142	78.7% (2,529/3,215)
Hepatitis B virus	Serum	560500	2	2.66806E-54	9.3% (299/3,215)
Hepatitis B virus	Serum	649082	2	1.08203E-30	5% (175/3,215)
Hepatitis B virus	Serum	298130	22918	7.56657E-56	100% (3,215)
Hepatitis B virus	Serum	352212	982	6.7019E-158	95.6% (3,074/3,215)
Hepatitis B virus	Serum	438146	18364	3.42494E-56	100% (3,215)

Hepatitis B virus	Serum	54750	1732	5.6804E-124	100% (3,215)
Hepatitis B virus	Serum	374364	477	6.3359E-179	90.6% (2,914/3,215)
Hepatitis B virus	Serum	320784	75	0	84% (2,709/3,215)
Hepatitis C virus	Serum	293316	5342	1.40256E-56	98.6% (9,171/9,297)
Hepatitis C virus	Serum	231960	307	2.06237E-50	74.6% (6,984/9,358)
Human coronavirus	Swabs	1203206	4	1.86221E-28	1.1% (328/30,521)
Human herpesvirus 6	Swabs	1050246	16	8.72727E-53	0.8% (1,309/161,296)
Human immunodeficiency virus	Serum	293316	355	8.4618E-102	55.1% (4,883/8,860)
Human mastadenovirus	Swabs	855702	6	5.02331E-30	1.6% (582/35,831)
Human mastadenovirus	Swabs	975450	287	0	36.2% (12,774/35,265)
Human metapneumovirus	Swabs	825274	522	0	74.5% (9,932/13,327)
Human parainfluenza virus	Swabs	701436	3	8.16848E-51	2.5% (393/15,502)
Human parainfluenza virus	Swabs	111152	427	0	59.0% (9,047/15,335)
Human parechovirus	Serum	331722	58	0	15.8% (1,155/7,320)
Human parechovirus	Stool	608352	52	7.29444E-96	16.2% (1,186/7,320)
Human pegivirus 2	Serum	293316	273	0	33.9% (3,237/9,538)
Human respiratory syncytial virus	Swabs	504418	28422	0	99.3% (15,165/15,276)
Human respiratory syncytial virus	Swabs	452112	9	3.04048E-35	3% (467/15,232)
Human rhinovirus	Serum	513280	483	0	59.4% (4,217/7,099)
Human rhinovirus	Swabs	811032	401	0	14.6% (974/6,692bp)
Human rhinovirus	Swabs	408734	8	3.66242E-55	5.4% (387/7,208bp)
Human rhinovirus	Swabs	489110	39	5.99188E-55	25.0% (1,761/7,047)
Human rhinovirus	Swabs	876786	67	0	61.8% (4,390/7,104)
Influenza A virus	Swabs	493794	23	3.39944E-53	5.2% (710/13,500)
Influenza B virus	Swabs	479434	594	6.7272E-145	58.4% (1,025/1,755)
Measles virus	Stool	441248	19530	0	96.6% (15,360/15,894)
Measles virus	Stool	435356	4	2.60588E-49	3.8% (602/15,894)
Rotavirus A	Serum	960504	2	5.84582E-25	<1% (155/18,550)
Rotavirus A	Serum	590870	2	1.59659E-12	<1% (100/18,550)
Rotavirus A	Serum	491942	366	0	83.0% (2,731/3,292)
Saffold virus	Swabs	1050246	29	4.5541E-138	9.2% (737/8,054)
Salivirus A	Stool	435356	4	1.95271E-37	7.3% (582/8,021)
WU Polyomavirus	Swabs	459132	164	1.1621E-129	45.3% (2,367/5,229)

REFERENCES

1. Thaipadunpanit J, Chierakul W, Wuthiekanun V, Limmathurotsakul D, Amornchai P, Boonslip S, Smythe LD, Limpaiboon R, Hoffmaster AR, Day NPJ, Peacock SJ. 2011. Diagnostic accuracy of real-time PCR assays targeting 16S rRNA and lipl32 genes for human leptospirosis in Thailand: A case-control study. PLoS One 6:1–6.

2. Laras K, Cao bao van, Bounlu K, Nguyen TKT, Olson JG, Thongchanh S, Tran NVA, Hoang KL, Punjabi N, Ha BK, An US, Insisiengmay S, Watts DM, Beecham HJ, Corwin AL. 2002. The importance of tourism in South-East Asia. Am J Trop Med Hyg 67:278–286.

3. Blacksell SD, Jenjaroen K, Phetsouvanh R, Wuthiekanun V, Day NPJ, Newton PN, Ching W-M. 2010. Accuracy of AccessBio Immunoglobulin M and Total Antibody Rapid Immunochromatographic Assays for the Diagnosis of Acute Scrub Typhus Infection. Clin Vaccine Immunol 17:263–6.

4. Jiang J, Chan T-C, Temenak JJ, Dasch GA, Ching W-M, Richards AL. 2004. Development of a quantitative real-time polymerase chain reaction assay specific for Orientia tsutsugamushi. Am J Trop Med Hyg 70:351–6.

5. Henry KM, Jiang J, Rozmajzl PJ, Azad AF, Macaluso KR, Richards AL. 2007. Development of quantitative real-time PCR assays to detect Rickettsia typhi and Rickettsia felis, the causative agents of murine typhus and flea-borne spotted fever. Mol Cell Probes 21:17–23.

6. Cherkaoui A, Emonet S, Ceroni D, Candolfi B, Hibbs J, Francois P, Schrenzel J. 2009. Development and validation of a modified broad-range 16S rDNA PCR for diagnostic purposes in clinical microbiology. J Microbiol Methods 79:227–31.

7. Weisburg WG, Barns SM, Pelletier DA, Lane DJ. 1991. 16S ribosomal DNA amplification for phylogenetic study. J Bacteriol 173:697–703.

8. Klempa B, Fichet-Calvet E, Lecompte E, Auste B, Aniskin V, Meisel H, Denys C,

Koivogui L, ter Meulen J, Krüger DH. 2006. Hantavirus in African wood mouse, Guinea. Emerg Infect Dis 12:838–40.

9. Cardosa MJ, Wang SM, Sum MSH, Tio PH. 2002. Antibodies against prM protein distinguish between previous infection with dengue and Japanese encephalitis viruses. BMC Microbiol 2:9.

10. Hue KDT, Tuan TV, Thi HTN, Bich CTN, Anh HH Le, Wills BA, Simmons CP. 2011. Validation of an internally controlled one-step real-time multiplex RT-PCR assay for the detection and quantitation of dengue virus RNA in plasma. J Virol Methods 177:168–73.

11. van Doornum GJJ, Guldemeester J, Osterhaus ADME, Niesters HGM. 2003. Diagnosing herpesvirus infections by real-time amplification and rapid culture. J Clin Microbiol 41:576–80.

12. de Jong MD, Weel JF, Schuurman T, Wertheim-van Dillen PM, Boom R. 2000. Quantitation of varicella-zoster virus DNA in whole blood, plasma, and serum by PCR and electrochemiluminescence. J Clin Microbiol 38:2568–73.

13. Beld M, Minnaar R, Weel J, Sol C, Damen M, van der Avoort H, Wertheim-van Dillen P, van Breda A, Boom R. 2004. Highly sensitive assay for detection of enterovirus in clinical specimens by reverse transcription-PCR with an armored RNA internal control. J Clin Microbiol 42:3059–64.

14. Jansen RR, Schinkel J, Koekkoek S, Pajkrt D, Beld M, de Jong MD, Molenkamp R. 2011. Development and evaluation of a four-tube real time multiplex PCR assay covering fourteen respiratory viruses, and comparison to its corresponding single target counterparts. J Clin Virol 51:179–85.

15. Corless CE, Guiver M, Borrow R, Edwards-Jones V, Fox AJ, Kaczmarski EB. 2001. Simultaneous detection of Neisseria meningitidis, Haemophilus influenzae, and Streptococcus

pneumoniae in suspected cases of meningitis and septicemia using real-time PCR. J Clin Microbiol 39:1553–8.

16. Nga TVT, Nghia HDT, Tu LTP, Diep TS, Mai NTH, Chau TTH, Sinh DX, Phu NH, Nga TTT, Chau NVV, Campbell J, Hoa NT, Chinh NT, Hien TT, Farrar J, Schultsz C. 2011. Real-time PCR for detection of Streptococcus suis serotype 2 in cerebrospinal fluid of human patients with meningitis. Diagn Microbiol Infect Dis 70:461–7.

17. Heddema ER, Beld MGHM, de Wever B, Langerak AAJ, Pannekoek Y, Duim B. 2006. Development of an internally controlled real-time PCR assay for detection of Chlamydophila psittaci in the LightCycler 2.0 system. Clin Microbiol Infect 12:571–5.

18. Pitcher D, Chalker VJ, Sheppard C, George RC, Harrison TG. 2006. Real-time detection of Mycoplasma pneumoniae in respiratory samples with an internal processing control. J Med Microbiol 55:149–55.

19. Reischl U, Lehn N, Sanden GN, Loeffelholz MJ. 2001. Real-time PCR assay targeting IS481 of Bordetella pertussis and molecular basis for detecting Bordetella holmesii. J Clin Microbiol 39:1963–6.

20. Logan C, O'Leary JJ, O'Sullivan N. 2006. Real-time reverse transcription-PCR for detection of rotavirus and adenovirus as causative agents of acute viral gastroenteritis in children. J Clin Microbiol 44:3189–95.

21. Logan C, O'Leary JJ, O'Sullivan N. 2007. Real-time reverse transcription PCR detection of norovirus, sapovirus and astrovirus as causative agents of acute viral gastroenteritis. J Virol Methods 146:36–44.

22. Dung TTN, Phat VV, Nga TVT, My PVT, Duy PT, Campbell JI, Thuy CT, Hoang NVM, Van Minh P, Le Phuc H, Tuyet PTN, Vinh H, Kien DTH, Huy HLA, Vinh NT, Nga TTT, Hau

NTT, Chinh NT, Thuong TC, Tuan HM, Simmons C, Farrar JJ, Baker S. 2013. The validation and utility of a quantitative one-step multiplex RT real-time PCR targeting rotavirus A and norovirus. J Virol Methods 187:138–43.

23. Hummel KB, Lowe L, Bellini WJ, Rota PA. 2006. Development of quantitative gene-specific real-time RT-PCR assays for the detection of measles virus in clinical specimens. J Virol Methods 132:166–173.

24. Dung TTN, Phat VV, Nga TVT, My PVT, Duy PT, Campbell JI, Thuy CT, Hoang NVM, Van Minh P, Le Phuc H, Tuyet PTN, Vinh H, Kien DTH, Huy HLA, Vinh NT, Nga TTT, Hau NTT, Chinh NT, Thuong TC, Tuan HM, Simmons C, Farrar JJ, Baker S. 2013. The validation and utility of a quantitative one-step multiplex RT real-time PCR targeting Rotavirus A and Norovirus. J Virol Methods 187:138–143.

25. Thanh T, Anh N, Tham N, Van H, Sabanathan S, Qui P, Ngan T, Van T, Nguyet L, Ny N, Thanh L, Chai O, Perera D, Viet D, Khanh T, Ha D, Tuan H, Wong K, Hung N, Chau N, Thwaites G, van Doorn H, Van Tan L. 2015. Validation and utilization of an internally controlled multiplex Real-time RT-PCR assay for simultaneous detection of enteroviruses and enterovirus A71 associated with hand foot and mouth disease. Virol J 12:85.

26. Frentiu FD, Zakir T, Walker T, Popovici J, Pyke AT, van den Hurk A, McGraw EA, O'Neill SL. 2014. Limited Dengue Virus Replication in Field-Collected Aedes aegypti Mosquitoes Infected with Wolbachia. PLoS Negl Trop Dis 8:1–10.

27. Hue KDT, Tuan TV, Thi HTN, Bich CTN, Anh HH Le, Wills BA, Simmons CP. 2011. Validation of an internally controlled one-step real-time multiplex RT-PCR assay for the detection and quantitation of dengue virus RNA in plasma. J Virol Methods 177:168–173.

28. Chook JB, Ong LY, Takebe Y, Chan KG, Choo M, Kamarulzaman A, Tee KK. 2015.

Molecular detection of HIV-1 subtype B, CRF01-AE, CRF33-01B, and newly emerging recombinant lineages in Malaysia. Am J Trop Med Hyg 92:507–512.

29. Jansen RR, Schinkel J, Koekkoek S, Pajkrt D, Beld M, Jong MD d., Molenkamp R. 2011. Development and evaluation of a four-tube real time multiplex PCR assay covering fourteen respiratory viruses, and comparison to its corresponding single target counterparts. J Clin Virol 51:179–185.

30. Harvala H, Robertson I, McWilliam Leitch EC, Benschop K, Wolthers KC, Templeton K, Simmonds P. 2008. Epidemiology and clinical associations of human parechovirus respiratory infections. J Clin Microbiol 46:3446–3453.

31. Drexler JF, De Souza Luna LK, Stöcker A, Silva Almeida P, Medrado Ribeiro TC, Petersen N, Herzog P, Pedroso C, Huppertz HI, Da Costa Ribeiro H, Baumgarte S, Drosten C. 2008. Circulation of 3 lineages of a novel saffold cardiovirus in humans. Emerg Infect Dis 14:1398–1405.

32. Haramoto E, Kitajima M, Otagiri M. 2013. Development of a reverse transcription-quantitative PCR assay for detection of salivirus/klassevirus. Appl Environ Microbiol 79:3529–3532.

33. Kuypers J, Campbell AP, Guthrie KA, Wright NL, Englund JA, Corey L, Boeckh M. 2012. WU and KI polyomaviruses in respiratory samples from allogeneic hematopoietic cell transplant recipients. Emerg Infect Dis 18:1580–1588.

34. N. T, H. K, Y. H, K. K, T. Y, Y. A, K. H, S. K. 2000. Monitoring four herpesviruses in unrelated cord blood transplantation. Bone Marrow Transplant 26:1193–1197.

35. Boom R, Sol CJA, Schuurman T, Breda A Van, Weel JFL, Beld M, Berge IJM, Dillen PMEW, Jong MD De. 2002. Human Cytomegalovirus DNA in Plasma and Serum Specimens of

Renal Transplant Recipients Is Highly Fragmented. J Clin Microbiol 40:4105–4113.

36. Phan TG, Mori D, Deng X, Rajidrajith S, Ranawaka U, Fan Ng TF, Bucardo-Rivera F, Orlandi P, Ahmed K, Delwart E. 2015. Small viral genomes in unexplained cases of human encephalitis, diarrhea, and in untreated sewage. Virology 482:98–104.

37. Tan L Van, Doorn HR Van, Trung D, Hong T, Phuong T, Vries M De. 2013. Identification of a New Cyclovirus in Cerebrospinal Fluid of Patients with Acute Central Nervus System Infections. MBio 4:1–10.

38. Frankel M, Forberg K, Coller KE, Berg MG, Hackett J, Cloherty G, Dawson GJ. 2017. Development of a high-throughput multiplexed real time RT-PCR assay for detection of human pegivirus 1 and 2. J Virol Methods 241:34–40.

39. Ursu K, Harrach B, Matiz K, Benko M. 2004. DNA sequencing and analysis of the right-hand part of the genome of the unique bovine adenovirus type 10. J Gen Virol 85:593–601.

40. Woo PCY, Lau SKP, Lam CSF, Lai KKY, Huang Y, Lee P, Luk GSM, Dyrting KC, Chan K-H, Yuen K-Y. 2009. Comparative Analysis of Complete Genome Sequences of Three Avian Coronaviruses Reveals a Novel Group 3c Coronavirus. J Virol 83:908–917.

41. Li L, Kapoor A, Slikas B, Bamidele OS, Wang C, Shaukat S, Masroor MA, Wilson ML, Ndjango J-BN, Peeters M, Gross-Camp ND, Muller MN, Hahn BH, Wolfe ND, Triki H, Bartkus J, Zaidi SZ, Delwart E. 2010. Multiple Diverse Circoviruses Infect Farm Animals and Are Commonly Found in Human and Chimpanzee Feces. J Virol 84:1674–1682.

42. Bailey L, Newman JF., Porterfield JS. 1966. The Multiplication of Nodamura Virus in Insect and Mammalian Cell Cultures. J Gen Virol 26:15–20.

43. Hansen TA, Mollerup S, Nguyen NP, White NE, Coghlan M, Alquezar-Planas DE, Joshi T, Jensen RH, Fridholm H, Kjartansdóttir KR, Mourier T, Warnow T, Belsham GJ, Bunce M,

Willerslev E, Nielsen LP, Vinner L, Hansen AJ. 2016. High diversity of picornaviruses in rats from different continents revealed by deep sequencing. Emerg Microbes Infect 5:e90.

44. Woo PCY, Lau SKP, Teng JLL, Tsang AKL, Joseph M, Wong EYM, Tang Y, Sivakumar S, Bai R, Wernery R, Wernery U, Yuen KY. 2014. Metagenomic analysis of viromes of dromedary camel fecal samples reveals large number and high diversity of circoviruses and picobirnaviruses. Virology 471–473:117–125.

45. Wu Z, Yang L, Ren X, He G, Zhang J, Yang J, Qian Z, Dong J, Sun L, Zhu Y, Du J, Yang F, Zhang S, Jin Q. 2016. Deciphering the bat virome catalog to better understand the ecological diversity of bat viruses and the bat origin of emerging infectious diseases. ISME J 10:609–620.

46. Xiao P, Li C, Zhang Y, Han J, Guo X, Xie L, Tian M, Li Y, Wang M, Liu H, Ren J, Zhou H, Lu H, Jin N. 2018. Metagenomic Sequencing From Mosquitoes in China Reveals a Variety of Insect and Human Viruses. Front Cell Infect Microbiol 8:1–11.

47. Phan TG, del Valle Mendoza J, Sadeghi M, Altan E, Deng X, Delwart E. 2018. Sera of Peruvians with fever of unknown origins include viral nucleic acids from non-vertebrate hosts. Virus Genes 54:33–40.

48. Naccache SN, Greninger AL, Lee D, Coffey LL, Phan T, Rein-Weston A, Aronsohn A, Hackett J, Delwart EL, Chiu CY. 2013. The Perils of Pathogen Discovery: Origin of a Novel Parvovirus-Like Hybrid Genome Traced to Nucleic Acid Extraction Spin Columns. J Virol 87:11966–11977.

49. Brito F, Cordey S, Delwart E, Deng X, Tirefort D, Lemoine-Chaduc C, Zdobnov E, Lecompte T, Kaiser L, Waldvogel-Abramowski S, Preynat-Seauve O. 2018. Metagenomics analysis of the virome of 300 concentrates from a Swiss platelet bank. Vox Sang 113:601–604.

50. Ngoi CN, Siqueira J, Li L, Deng X, Mugo P, Graham SM, Price MA, Sanders EJ, Delwart E. 2016. The plasma virome of febrile adult kenyans shows frequent parvovirus B19 infections and a novel arbovirus (Kadipiro virus). J Gen Virol 97:3359–3367.

51. Aguiar ERGR, Olmo RP, Paro S, Ferreira FV, De Faria IJDS, Todjro YMH, Lobo FP, Kroon EG, Meignin C, Gatherer D, Imler JL, Marques JT. 2015. Sequence-independent characterization of viruses based on the pattern of viral small RNAs produced by the host. Nucleic Acids Res 43:6191–6206.

52. Belaganahalli MN, Maan S, Maan NS, Nomikou K, Pritchard I, Lunt R, Kirkland PD, Attoui H, Brownlie J, Mertens PPC. 2012. Full genome sequencing and genetic characterization of eubenangee viruses identify pata virus as a distinct species within the genus orbivirus. PLoS One 7.

53. Horta AB, Ardisson-Araujo DMP, da Silva LA, de Melo FL, da Silva Morgado F, Franco Lemos MV, Ribeiro ZA, Boiça AL, Wilcken CF, Ribeiro BM. 2018. Genomic analysis of a cypovirus isolated from the eucalyptus brown looper, Thyrinteina arnobia (Stoll, 1782) (Lepidoptera: Geometridae). Virus Res 253:62–67.

54. Medeiros DB de A, Diniz Júnior JAP, Cardoso JF, Silva SP, da Silva DEA, de Oliveira LF, E Vasconcelos JM, Chiang JO, Dias AA, Nunes MRT, Vianez Júnior JL da SG, Vasconcelos PFC. 2014. Nearly complete genome sequence of curionopolis virus, a culicoides-related rhabdovirus isolated in the brazilian Amazon region. Genome Announc 2:e01158-14.

55. Bruenn JA. 1993. A closely related group of RNA-dependent RNA polymerases from double-stranded RNA viruses. Nucleic Acids Res 21:5667–5669.

56. Taylor DJ, Ballinger MJ, Bowman SM, Bruenn JA. 2013. Virus-host co-evolution under a modified nuclear genetic code. PeerJ 1:e50.

57. Ai YP, Zhong J, Chen CY, Zhu HJ, Gao B Da. 2016. A novel single-stranded RNA virus isolated from the rice-pathogenic fungus Magnaporthe oryzae with similarity to members of the family Tombusviridae. Arch Virol 161:725–729.

58. Ng TFF, Marine R, Wang C, Simmonds P, Kapusinszky B, Bodhidatta L, Oderinde BS, Wommack KE, Delwart E. 2012. High Variety of Known and New RNA and DNA Viruses of Diverse Origins in Untreated Sewage. J Virol 86:12161–12175.
